# Epidural abscess and spondylitis caused by *Veillonella parvula* in a man on hemodialysis

**DOI:** 10.1002/ccr3.4660

**Published:** 2021-10-08

**Authors:** Masaru Kurihara, Itaru Tamaki, Yasuharu Tokuda

**Affiliations:** ^1^ Department of Hospital Medicine Urasoe General Hospital Okinawa Japan

**Keywords:** epidural abscess, Gram‐negative bacteria, hemodialysis, infectious diseases, opportunistic infections, *Veillonella* infections

## Abstract

*Veillonella* species rarely cause serious infections, but the incidence of infection has been increasing among immunocompromised individuals. This case of an epidural abscess and spondylitis caused by *Veillonella parvula* in a hemodialysis patient illustrates the importance of performing anaerobic blood culture in immunocompromised patients with signs of infection.

## Graphical abstract

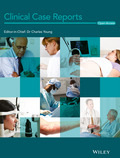

## 1 INTRODUCTION

*Veillonella* is an anaerobic gram‐negative coccus that inhabits the oral cavity, gastrointestinal tract, and female genital tract.^1^, ^2^, ^3^ Thirteen species of the genus have been identified, of which six species have been isolated from the human oral cavity.^4^ *Veillonella* species were reported coaggregate with *Streptococcus* spp^4^ and were formerly considered to be non‐pathogenic and to rarely cause serious infections, but in recent years, there has been an increasing number of reports of human infections among immunocompromised individuals.^5^ Here, we report a rare case of an epidural abscess and spondylitis caused by *Veillonella parvula*.

## 2 CASE HISTORY

A 52‐year‐old Japanese man presented to the emergency department of our hospital with a 1‐week history of low back pain. He had also developed difficulty in walking over the previous 2 days. He had a 10‐year history of end‐stage renal disease caused by glomerulonephritis and was on hemodialysis three times a week. He had smoked 20 cigarettes/day for 30 years and was an occasional drinker. On examination, he was alert, with a temperature of 37.0℃, blood pressure of 105/74 mm Hg, respiratory rate of 32 breaths/min, and heart rate of 180 beats/min. No abnormalities were detected on respiratory or cardiac examination. Abdominal examination showed tenderness over an area from the right buttock to the right thigh. There was no redness or tenderness at the dialysis shunt site, located on the left forearm. Laboratory test results on admission showed a white blood cell count of 16,200 cells/µL, hemoglobin level of 11.8 g/dl, and platelet count of 102,000/µl. His serum potassium (8.0 mmol/L) and C‐reactive protein (41.7 mg/dl) levels were elevated. His other blood test results are shown in Table 1.

**TABLE 1:** Laboratory data of the patient

Variable	Reference range	Value on admission
Red blood cell (per μl)	3860 000–4920 000	4620 000
Hemoglobin (g/dl)	11.6–14.8	11.8
Hematocrit (%)	35.1–44.4	37.7
White blood cell (per μl)	3300–8600	16200
Platelets (per μl)	158 000–348 000	1020000
Sodium (mmol/L)	135–145	136
Potassium (mmol/L)	3.6–4.8	8.0
Chloride (mmol/L)	101–108	100
Urea nitrogen (mg/dl)	8.0–20.0	80.6
Creatinine (mg/dl)	0.46–0.79	14.46
Alanine aminotransferase (U/L)	13–30	86
Aspartate aminotransferase (U/L)	7–23	21
Alkaline phosphatase (U/L)	106–322	378
C‐reactive protein (mg/dl)	0.0–0.1	41.7

### 2.1 Differential diagnosis, investigations, and treatment

**FIGURE 1:**
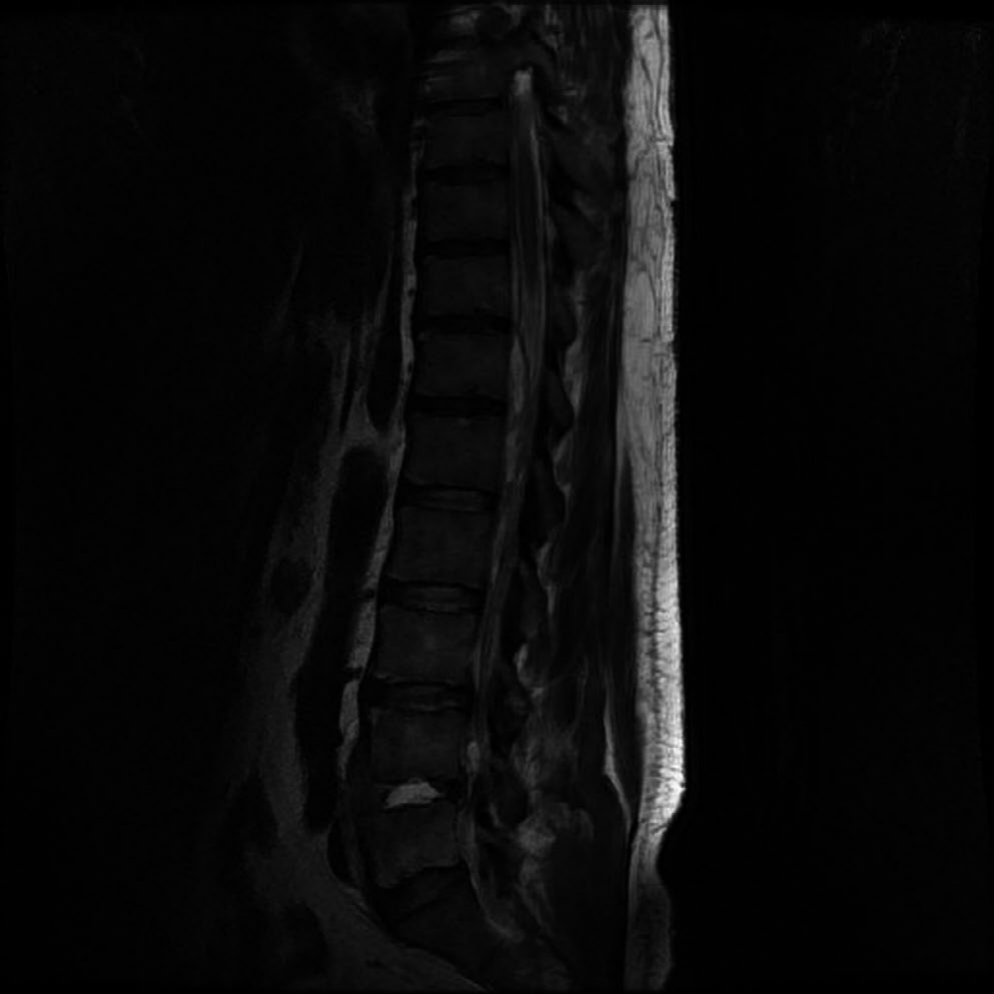
Lumbar magnetic resonance imaging on the second day of hospitalization. The image shows an extensive epidural abscess with evidence of discitis in the L5/S1 region

He was admitted to the hospital and underwent emergency hemodialysis to correct the hyperkalemia, and his serum potassium level decreased to 4.5 mmol/L. On day 2 of admission, he developed a fever (40.1℃), and his right lower back pain worsened. Pelvic magnetic resonance imaging (MRI) revealed findings suggestive of an epidural abscess and spondylitis at the L5/S1 intervertebral disk (Figure 1). One bottle of blood culture revealed anaerobic gram‐negative cocci, on day 3 of admission. Based on these findings, the patient's condition was diagnosed as spondylitis and epidural abscess caused by anaerobic bacteria. Antimicrobial therapy was initiated with intravenous ampicillin/sulbactam (3 g 6‐hourly). Testing using a VITEK‐ANC Card® identified the pathogen as a *Veillonella* species. and 16S rRNA polymerase chain reaction identified the species as *Veillonella parvula*. Based on the susceptibility by the micro liquid dilution method (Table 2), the intravenous antimicrobial agent was changed to ampicillin (2 g 6‐hourly).

**TABLE 2:** Antimicrobial susceptibility of this patient

Antimicrobial	Minimal inhibitory concentration (µg/ml)
Ampicillin	≤0.5
Piperacillin	8
Cefaclor	≤8
Cefotiam	16
Cefotaxime	16
Ceftazidime	≤1
Ceftriaxone	4
Flomoxef	≤1
Azithromycin	16
Imipenem and cilastatin	2
Ampicillin and sulbactam	≤0.5
Piperacillin and tazobactam	4
Gentamycin	>16
Clindamycin	>4
Minocycline	≤2
Levofloxacin	≤0.5

### 2.2 Outcome and follow‐up

After initiation of ampicillin, the clinical, laboratory, and imaging signs of inflammation gradually improved. Finally, the level of C‐reactive protein had fallen to 1.4 mg/dl on day 57. Antibiotics were administered for 58 days, and the patient was discharged on day 60. He has been followed up further for 6 months since his discharge and has not experienced a recurrence.

## 3 DISCUSSION

This is a rare case of epidural abscess and spondylitis caused by *V*. *parvula*. Hirai et al.^6^ reviewed the literature from 1976 to October 2015 and found 31 cases of *Veillonella* infection in humans. Of these cases, five were musculoskeletal infections caused by *V*. *parvula*, including four spinal infections. There has been only one case of an epidural abscess caused by *V*. *parvula* reported previously.^2^ To our knowledge, the current case is the first report of human *V*. *parvula* infection in Japan. The previously reported case of an epidural abscess caused by *V*. *parvula* ^2^ was in a cancer patient, and our patient was on hemodialysis as a risk factor; therefore, *V*. *parvula* appears to be an opportunistic pathogen that affects immunocompromised patients. Previous reports have indicated the importance of anaerobic culture for identifying this pathogen^6^; in our case, anaerobic culture isolated the organism, and further investigation revealed the species, suggesting that anaerobic culture should be considered in patients with signs of an epidural abscess, especially in immunocompromised patients. On the other hand, blood tests are used for long‐term management. As had done in previous reports,^7^, ^8^ we followed the inflammatory marker to determine the duration of treatment.

Most *Veillonella* spinal infections reported to date have been associated with a subacute course of lower back pain from 1 week to 4 months.^1^, ^7^, ^8^ Spinal infections may develop serious complications if the diagnosis is delayed.^9^ Our case was diagnosed early using MRI. MRI should be considered in immunocompromised patients with signs of musculoskeletal infection with low back pain, such as our case.

In conclusion, this is the first case of epidural abscess and spondylitis caused by *V*. *parvula* reported in Japan. Musculoskeletal infection caused by *Veillonella* can lead to chronic back pain in immunocompromised patients, and early diagnosis using MRI and anaerobic culture are recommended.

## CONFLICT OF INTEREST

None declared.

## AUTHOR CONTRIBUTIONS

All authors critically revised the report, commented on drafts of the manuscript, and approved the final report.

## CONSENT FOR PUBLICATION

The patient has provided informed consent for publication.

## Data Availability

The data that support the findings of this study are available from the corresponding author, MK, upon reasonable request.
